# Small-vessel-disease-induced white matter damage in occipital lobe epilepsy

**DOI:** 10.3389/fneur.2025.1538598

**Published:** 2025-02-11

**Authors:** Jinseung Kim, Dong Ah Lee, Ho-Joon Lee, Kang Min Park

**Affiliations:** ^1^Department of Family Medicine, Busan Paik Hospital, Inje University College of Medicine, Busan, Republic of Korea; ^2^Department of Neurology, Haeundae Paik Hospital, Inje University College of Medicine, Busan, Republic of Korea; ^3^Department of Radiology, Haeundae Paik Hospital, Inje University College of Medicine, Busan, Republic of Korea

**Keywords:** epilepsy, diffusion tensor imaging, cerebral small vessel diseases, white matter, neuroimaging

## Abstract

**Background:**

Peak width of skeletonized mean diffusivity (PSMD) is a novel marker of small vessel disease. This study aimed to investigate the presence of small vessel disease in patients with occipital lobe epilepsy (OLE) using PSMD.

**Methods:**

We enrolled 27 patients newly diagnosed with OLE and included 29 healthy controls. The age and sex of the patients and controls were comparable. Diffusion tensor imaging (DTI) was performed using a 3 T MRI scanner. We measured the PSMD based on DTI in several steps, including preprocessing, skeletonization, application of a custom mask, and histogram analysis, using the FSL program. We compared PSMD between patients with OLE and healthy controls. Additionally, we performed a correlation analysis between PSMD and clinical factors in patients with OLE.

**Results:**

Our findings revealed that the patients with OLE exhibited higher PSMD compared to healthy controls (2.459 vs. 2.079 × 10^−4^ mm^2^/s, *p* < 0.001). In addition, PSMD positively correlated with age (*r* = 0.412, *p* = 0.032). However, the PSMD of the patients with OLE was not associated with other clinical factors such as age at seizure onset and duration of epilepsy.

**Conclusion:**

We demonstrated that patients with OLE had a higher PSMD than healthy controls, indicating evidence of small vessel disease in patients with OLE. This finding also highlights the potential of PSMD as a marker for detecting small vessel diseases in epileptic disorders.

## 1 Introduction

Occipital lobe epilepsy (OLE) is a relatively uncommon type of focal epilepsy, originating in the occipital lobe and accounting for approximately 2–8% of surgical cases (1). It can result from structural brain abnormalities, such as tumors, strokes, or hemorrhages. However, it also occurs as part of self-limited focal epilepsies in childhood, including self-limited epilepsy with autonomic seizures or childhood occipital visual epilepsy (2).

Diffusion tensor imaging (DTI) is a magnetic resonance imaging (MRI) sequence that measures the diffusion of water molecules in tissues. Traditionally, DTI has been utilized in epilepsy surgery to define surgical margins using tractography (3). It also provides valuable insight into the microstructural integrity of white matter tracts, which cannot be visualized with conventional brain MRI (4, 5). DTI can facilitate calculations of fractional anisotropy (FA) and mean diffusivity (MD) values, which serve as indicators of white matter microstructure. In patients with focal epilepsy, FA values generally increase, while MD values tend to decrease compared to healthy controls, with more pronounced changes observed on the ipsilateral side than the contralateral side (6). In addition, DTI can be used to investigate the structural connectivity of the brain. In patients with OLE, global integration is reduced, and alterations in local networks beyond the occipital lobe have been observed (7). Recently, DTI has been used to investigate the glymphatic system function of the brain, with dysfunction in this system identified in patients with OLE (8). Therefore, DTI is increasingly being used in both research and clinical practice, particularly for patients with epilepsy, including those with OLE.

Peak width of skeletonized mean diffusivity (PSMD) is a recently proposed neuroimaging marker derived from DTI that serves as an objective index for quantifying white matter damage caused by small vessel disease (9, 10). PSMD can be fully automatically calculated in a short time and has shown a stronger correlation with cognitive impairment than conventional DTI measures such as FA or MD. As a result, active research using PSMD has been conducted in various neurological diseases, such as multiple sclerosis, stroke, cerebral amyloid angiopathy, and dementia (11–15). However, white matter damage due to small vessel disease in patients with epilepsy, particularly OLE, has never been studied using PSMD.

Therefore, in this study, we aimed to investigate the degree of white matter damage due to small vessel disease in patients with OLE compared to healthy controls using PSMD. Additionally, we investigated the volumes of white matter hypointensities, which is another MRI marker for white matter damage based on T1-weighted imaging, in patients with OLE and compared them with healthy controls. We hypothesized that white matter damage in patients with OLE might be associated with small vessel disease than in the healthy control group.

## 2 Methods

### 2.1 Participants

This study was approved by the Institutional Review Board, and informed consent was obtained from all participants. We enrolled 27 patients newly diagnosed with OLE according to the ILAE criteria (16, 17). Only patients whose ictal semiology clearly indicated OLE and whose electroencephalography showed ictal or interictal epileptiform discharges originating in the occipital lobe were included in this study. DTI and T1-weighted MRI was performed at the time of OLE diagnosis in the drug-naïve state. We excluded the following participants from this study: (1) those with structural lesions on brain MRI that could influence the results of imaging analysis, (2) those with any neurological diseases other than OLE, (3) those with risk factors for small vessel disease, such as diabetes, hypertension, or dyslipidemia, or (4) those who did not consent to participate in the study. We also enrolled 29 age- and sex-matched healthy controls who had not been diagnosed with any medical or neurological diseases. The healthy controls underwent DTI and T1-weighted MRI, and their brain MRI revealed no structural lesions. Like the patients, the healthy controls did not have the risk factors associated with small vessel disease.

### 2.2 DTI scan

All DTI and T1-weighted MRI scans were performed using a 3.0 T MRI scanner (AchievaTx; Phillips Healthcare, Best, Netherlands) equipped with a 32-channel head coil for both patients with OLE and healthy controls. The DTI scans utilized spin-echo single-shot echo-planar pulse sequences with 32 different diffusion directions (repetition time/echo time, 8,620/85 ms; flip angle, 90°; slice thickness, 2.25 mm, acquisition matrix, 120 × 120; field of view, 240 × 240 mm^2^; and *b*-value, 1,000 s/mm^2^). The three-dimensional T1-weighted images were scanned using the following parameters: inversion time = 1,300 ms, repetition time/echo time = 8.6/3.96 ms, flip angle = 8°, and isotropic voxel size = 1 mm^3^.

### 2.3 Obtaining the PSMD

**Figure 1:**
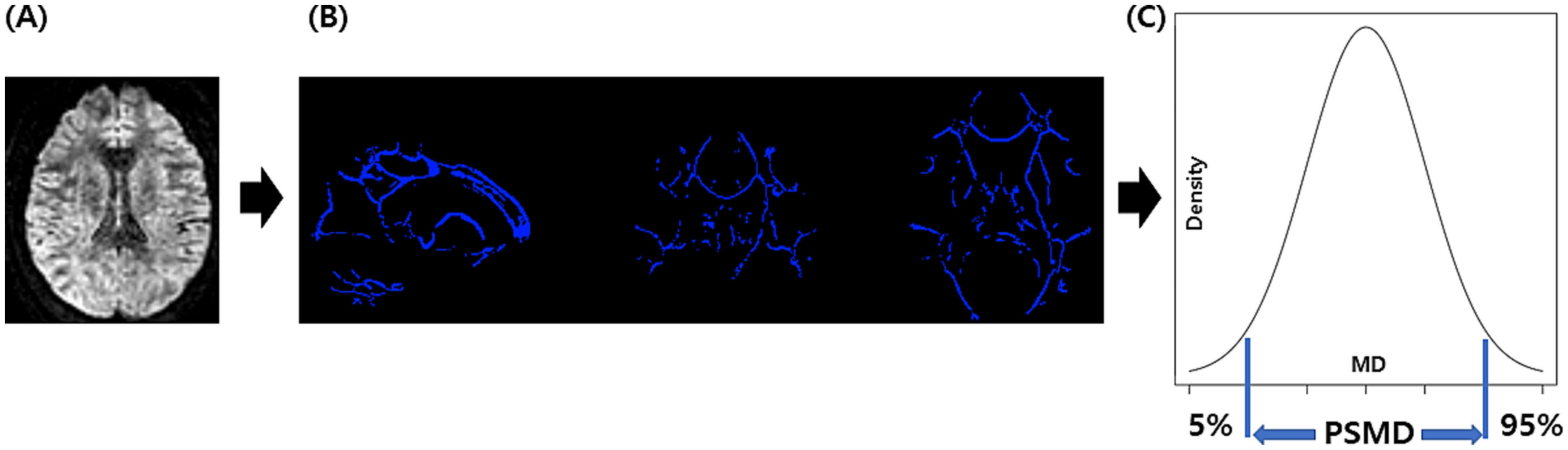
The process for obtaining PSMD: we perform DTI acquisition on the participants, followed by preprocessing steps including motion and eddy current correction, brain extraction, and tensor fitting **(A)**. Subsequently, we conducted skeletonization, which included normalization, projection onto the skeleton template, and the application of a custom mask **(B)**. Finally, we performed histogram analysis and calculated PSMD based on the difference between the 95th and 5th percentiles **(C)**. PSMD, peak width of skeletonized mean diffusivity.

Figure 1 shows the process for obtaining PSMD from DTI using the FSL program installed on a Linux system, involving a total of four steps (9, 10). The first step preprocesses the DTI, which includes motion and eddy current correction, brain extraction, and tensor fitting. The second step is skeletonization, which involves tract-based spatial statistics obtained by registering an FA map to the common space and projecting it onto the skeleton. The same transformation matrices were used for MD data to obtain a skeletonized MD map. The third step was the application of a custom mask using the template thresholded at an FA value of 0.3 and a custom-made mask. The fourth step was histogram analysis, in which the width of the histogram (the difference between 95 and 5) derived from the MD values of all voxels included in the skeleton was obtained.

### 2.4 White matter hypointensities segmentation

To segment white matter hypointensities from T1-weighted images and acquire the volumes of white matter hypointensities, we used WMH-SynthSeg (18), which provides segmentation for white matter hyper- or hypointensities from scans of any resolution and contrast without retraining, available as module in the development version of Freesurfer.

### 2.5 Statistical analysis

An independent sample *t*-test was used to compare age and PSMD values between patients with OLE and healthy controls. The Mann–Whitney test was used to compare the volumes of white matter hypointensities between the groups. The chi-square test was used to compare sex differences between the groups. Pearson’s correlation test was used for correlation analysis. The performance of the classification was evaluated using the receiver operating characteristic (ROC) curve analysis. Statistical significance was considered when the *p*-value was less at *p* < 0.05. All statistical analyses were performed using MedCalc^®^ Statistical Software version 22.009 (MedCalc Software Ltd., Ostend, Belgium; https://www.medcalc.org; 2023).

## 3 Results

### 3.1 Demographic and clinical characteristics of participants

Table 1 shows the demographic data in the participants and clinical characteristics of patients with OLE. There were no significant differences in age or sex between the OLE patients and the healthy control group.

**Table 1:** Demographic data in participants and clinical characteristics of patients with OLE.

	Patients with JME (*N* = 27)	Healthy controls (*N* = 29)	*p*-value
Demographic data			
Age, years (SD)	34.2 (15.6)	32.8 (4.1)	0.627
Men, *N* (%)	12 (44.4)	11 (37.9)	0.623
Clinical data			
Age of seizure onset, years (SD)	16.5 (15.9)		
Duration of epilepsy, months (SD)	156.0 (154.4)		
Number of seizures prior to treatment	5 (2.3)		
Initial seizure semiology			
Visual symptoms, *N* (%)	18 (66.6)		
Oculomotor symptoms, *N* (%)	8 (29.6)		
Others, *N* (%)	1 (3.7)		

PSMD, peak width of skeletonized mean diffusivity; OLE, occipital lobe epilepsy; SD, standard deviation.

### 3.2 Difference in the PSMD between the groups

**Figure 2:**
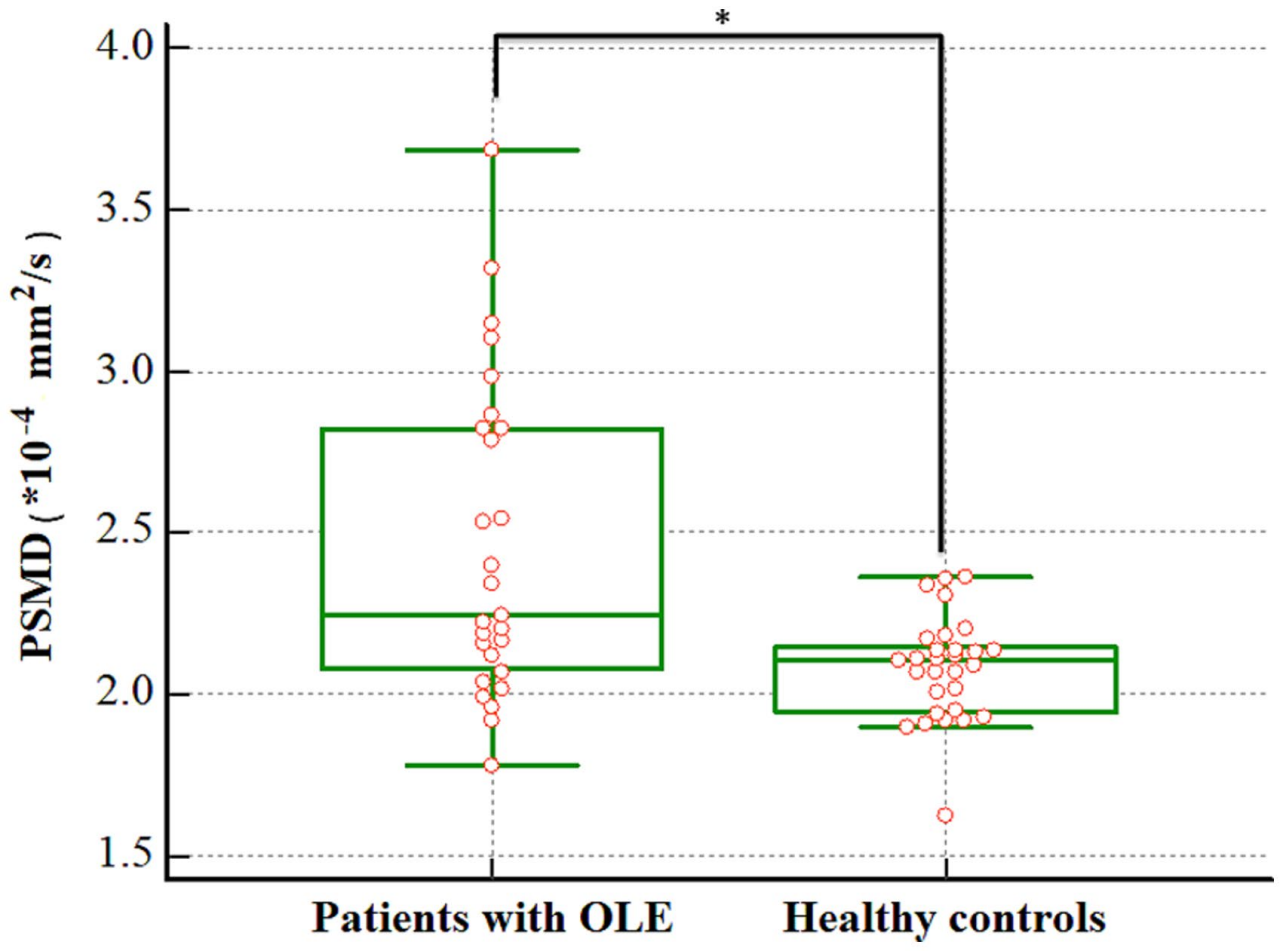
Difference in the PSMD between patients with OLE and healthy controls. The PSMD was higher in the OLE group than in the healthy control group (2.459 vs. 2.079 × 10^−4^ mm^2^/s, *p* < 0.001). PSMD, peak width of skeletonized mean diffusivity; OLE, occipital lobe epilepsy. ^*^p < 0.05.

There was a significant difference in the PSMD between patients with OLE and healthy controls. The patients with OLE exhibited higher PSMD compared to healthy controls (2.459 vs. 2.079 × 10^−4^ mm^2^/s, *p* < 0.001) (Figure 2).

### 3.3 Difference in the volumes of white matter hypointensities between the groups

The volumes of white matter hypointensities were higher in patients with OLE than that in the healthy controls [1309.6 (interquartile range, 1165.5–2793.2) vs. 1141.0 (interquartile range, 874.1–1298.9) mm^3^, *p* = 0.011].

### 3.4 ROC curve analysis

**Figure 3:**
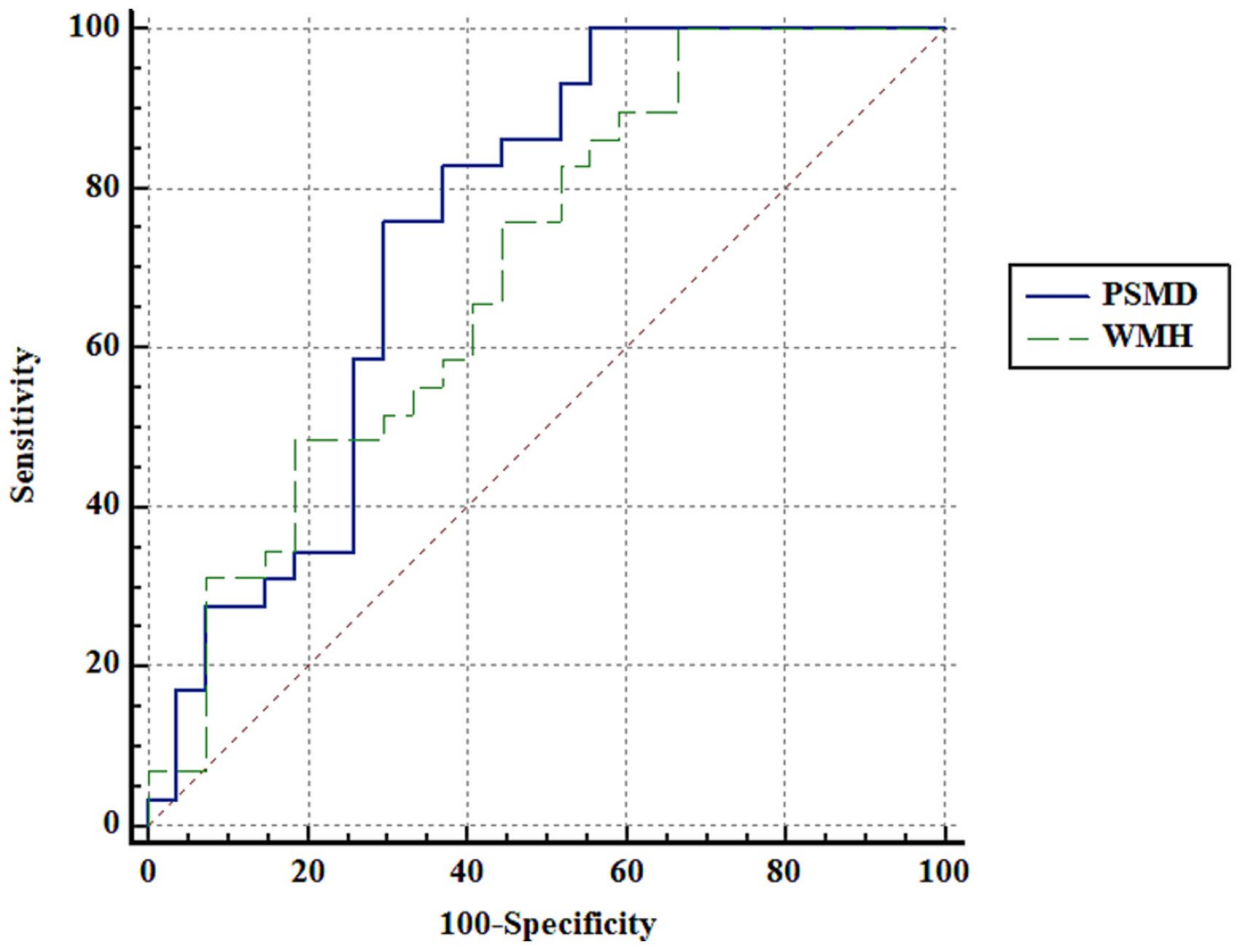
ROC curve analysis. ROC curve analysis using PSMD and the volumes of white matter hypointensities shows an area under curve (AUC) of 0.747 and 0.699, respectively, in distinguishing the patients with OLE and healthy controls. Although the AUC using PSMD is higher than that of the volumes of white matter hypointensities, there are no significant difference in the comparison of AUC between PSMD and the volumes of white matter hypointensities in distinguishing the groups (*p* = 0.602). PSMD, peak width of skeletonized mean diffusivity; WMH, the volumes of white matter hypointensities.

ROC curve analysis using PSMD showed an area under curve (AUC) of 0.747 in distinguishing the patients with OLE and healthy controls (*p* < 0.001). Additionally, ROC curve analysis using the volumes of white matter hypointensities revealed an AUC of 0.699 in distinguishing the groups (*p* = 0.005). Although the AUC using PSMD was higher than that of the volumes of white matter hypointensities, there were no significant difference in the comparison of AUC between PSMD and the volumes of white matter hypointensities in distinguishing the groups (*p* = 0.602) (Figure 3).

### 3.5 Correlation between the PSMD and clinical characteristics

**Figure 4:**
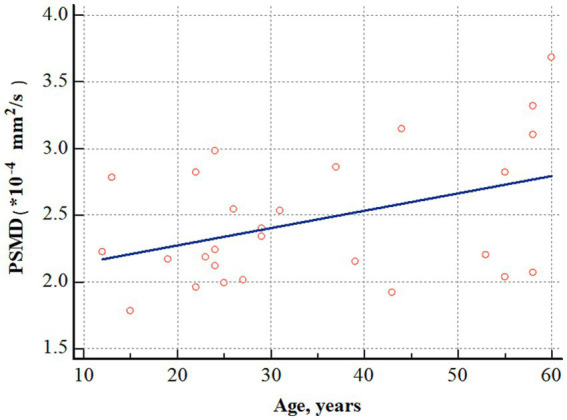
Correlation analysis between age and PSMD in patients with OLE. PSMD positively correlated with age (*r* = 0.412, *p* = 0.032). PSMD, peak width of skeletonized mean diffusivity; OLE, occipital lobe epilepsy.

In patients with OLE, a positive correlation was observed between PSMD and age (*r* = 0.412, *p* = 0.032) (Figure 4). However, PSMD was not associated with other clinical factors, such as age at seizure onset (*r* = 0.082, *p* = 0.695), duration of epilepsy (*r* = −0.127, *p* = 0.545), and number of seizures prior to treatment (*r* = 0.058, *p* = 0.770).

## 4 Discussion

This study is the first to demonstrate that PSMD is higher in patients with OLE than in healthy controls, indicating the presence of white matter damage due to small vessel disease in these patients. In addition, in patients with OLE, PSMD increases proportionally with age, confirming that small vessel disease progresses further with aging.

This study demonstrates the presence of white matter damage due to small vessel disease in patients with OLE, which aligns with previous studies. Maxwell et al. (19) investigated the presence of small vessel disease in 105 patients with epilepsy, particularly late-onset epilepsy, and 105 healthy controls. They used periventricular and subcortical white matter lesions as indicators of small vessel disease. They found that small vessel disease was present in 49.5% of patients with epilepsy, compared to 32.3% of the healthy controls, concluding that small vessel disease is more prevalent in individuals with epilepsy. Hanby et al. (19) analyzed white matter hyperintensities with automatic quantitation in patients with focal epilepsy and healthy controls, revealing higher white matter hyperintensities volume in patients with epilepsy (1,340 mm^3^) compared to controls (514 mm^3^) (20). Another study examined the correlation between the location of white matter lesions and the frequency of clinical symptoms such as stroke, seizure, vertigo, and gait apraxia. They reported that seizures were more frequent when lesions were located in the parieto-occipital lobe (21). This finding suggests an association between OLE and small vessel disease, similar to the present study. However, a previous study reported that epilepsy associated with leukoaraiosis was most closely related to the temporal lobe; therefore, further research is needed (22).

The cross-sectional design of this study makes it challenging to establish a cause-and-effect relationship between small vessel disease and OLE. Thus, two primary hypotheses were considered. The first hypothesis for the small vessel disease-OLE relationship is that small vessel disease may cause OLE. Previous animal experiments demonstrated in hypertensive rats that small vessel disease induced focal epilepsy more often than generalized epilepsy (23). It was observed that early treatment with enalapril could reduce the incidence of epilepsy in these animals, suggesting a role for small vessel disease in epileptogenesis (23). Additionally, studies on humans have demonstrated that hypertension is an independent risk factor for epilepsy (24, 25). Patients with hypertension are approximately twice as likely to develop epilepsy compared to those without, with those having uncontrolled hypertension being up to 10 times more likely to develop epilepsy (24, 25). Small vessel disease can cause endothelial dysfunction and blood-brain barrier leakage, leading to extravasation of serum proteins and inflammation, which may contribute to epileptogenesis. Diffuse cerebral microangiopathy can impair cerebral perfusion, leading to epileptogenesis via neurovascular uncoupling (26, 27).

Another hypothesis is that small vessel disease is caused by seizures that occur in patients with OLE. Recurrent seizures have shown to cause depolarization of pericytic mitochondria and subsequent vasoconstriction, resulting in small vessel disease, which is associated with impaired neurovascular coupling and increased blood-brain barrier permeability (28). Arteriole vasoconstriction, mediated by cyclooxygenase-2 and L-type calcium channels, plays an important role in hypoperfusion/hypoxia resulting from recurrent seizures (29). Furthermore, while the cerebral cortex is primarily involved in epileptic seizures, secondary changes in the cerebral white matter, including major association, commissural, and projection fibers, are well-documented. These changes are correlated with age of seizure onset and duration of epilepsy (30–32) and maybe induced by multiple mechanisms, including excitotoxicity with excessive glutamate release, inflammation response by microglia and astrocytes, oxidative stress by reactive oxygen species, and blood-brain barrier disruption (30–32).

We also confirmed that white matter damage due to small vessel disease worsened with age in patients with OLE. Several factors contribute to this worsening with age: endothelial dysfunction, atherosclerosis, increased reactive oxygen species, and chronic low-grade inflammation. The endothelium is located inside the blood vessels, and with aging, its function declines, making it difficult to maintain the integrity of the blood vessels and reducing its ability to regulate blood flow (33). Atherosclerosis is caused by the development of plaques in both large and small blood vessels. This plaque buildup impedes blood flow, increases vessel rigidity, and reduces perfusion (34). The increase in reactive oxygen species with age causes injury to the blood vessel walls (35). Finally, as we age, chronic low-grade inflammation occurs, which increases vessel stiffness and plaque formation, resulting in the destruction of the vascular endothelium (36). Through this study, we confirmed that small vessel disease worsens with age in patients with OLE, even in the absence of vascular risk factors, such as hypertension, diabetes, or dyslipidemia.

This study is the first to demonstrate white matter damage due to small vessel disease in patients with OLE. However, it has some limitations. The sample size, i.e., the number of patients enrolled in this study, was relatively small due to the rarity of OLE and the exclusion of patients with structural lesions that could affect DTI analysis. Additionally, to exclude the influence of anti-seizure medications on DTI measurements, only patients with their first diagnosis of epilepsy at the time of DTI imaging were enrolled. Another limitation of this study is the cross-sectional design, which did not allow us to establish a cause-and-effect relationship between small vessel disease and OLE. The study was conducted at a center specializing in epilepsy disorders; hence, the results cannot be generalized to all patients with epilepsy. In addition, the results were limited to OLE; therefore, further research with a larger sample size is needed for other focal epilepsy or generalized epilepsy. Lastly, we could not analyze the volumes of white matter hyperintensities, since some datasets lacked fluid attenuated inversion recovery images. Instead of the volumes of white matter hyperintensities, we analyzed the volumes of white matter hypointensites, which is another MRI marker for white matter damage based on T1-weighted imaging. Although the volumes of white matter hyperintensities are more accurate and widely used to assess white matter damage, they have strong correlation with the volumes of white matter hypointensites.

## 5 Conclusion

We demonstrated that patients with OLE had a higher PSMD than healthy controls, indicating the presence of small vessel disease in patients with OLE. This finding also highlights the potential of PSMD as a marker for detecting small vessel disease in epilepsy.

## Data Availability

The raw data supporting the conclusions of this article will be made available by the authors, without undue reservation.
